# Prevalence of blindness and distance vision impairment in the Gambia across three decades of eye health programming

**DOI:** 10.1136/bjophthalmol-2021-320008

**Published:** 2021-12-23

**Authors:** Abba Hydara, Islay Mactaggart, Suzannah J Bell, John A Okoh, Segun I Olaniyan, Mildred Aleser, Hannaa Bobat, Andy Cassels-Brown, Ben Kirkpatrick, Min J Kim, Ian McCormick, Hannah Faal, Matthew J Burton

**Affiliations:** 1 Sheikh Zayed Regional Eye Care Centre, Banjul, Gambia; 2 International Centre for Eye Health, London School of Hygiene & Tropical Medicine, London, UK; 3 Moorfields Eye Hospital NHS Foundation Trust, London, UK; 4 Department of Ophthalmology, University College Hospital Ibadan, Ibadan, Oyo, Nigeria; 5 St Mary's Hospital, Isle of Wight NHS Trust, Newport, Isle of Wight, UK; 6 The Fred Hollows Foundation, Sydney, New South Wales, Australia; 7 Department of Ophthalmology, NHS Highland, Inverness, UK; 8 Department of Ophthalmology, University of Calabar, Calabar, Cross River, Nigeria; 9 National Institute for Health Research Biomedical Research Centre for Ophthalmology, Moorfields Eye Hospital NHS Foundation Trust, London, UK

**Keywords:** epidemiology, public health, vision

## Abstract

**Background/aims:**

The 1986 Gambia National Eye Health Survey provided baseline data for a National Eye Health Programme. A second survey in 1996 evaluated changes in population eye health a decade later. We completed a third survey in 2019, to determine the current state of population eye health, considering service developments and demographic change.

**Methods:**

We estimated prevalence and causes of vision impairment (VI) in a nationally representative population-based sample of adults 35 years and older. We used multistage cluster random sampling to sample 10 800 adults 35 and above in 360 clusters of 30. We measured monocular distance visual acuity (uncorrected and with available correction) using Peek Acuity. Participants with either eye uncorrected or presenting (with available correction) acuity <6/12 were retested with pinhole and refraction, and dilated exams were completed on all eyes by ophthalmologists using a direct ophthalmoscope, slit lamp and 90 D lens.

**Results:**

We examined 9188 participants (response rate 83%). The 2013 census age–sex adjusted prevalence of blindness (presenting acuity<3/60 in better seeing eye) was 1.2% (95% CI 0.9 to 1.4) and of moderate or severe VI (MSVI,<6/18 to ≥3/60) was 8.9% (95% CI 9.1 to 9.7). Prevalence of all distance VI (<6/12) was 13.4% (12.4–14.4). Compared with 1996, the relative risk of blindness decreased (risk ratio 0.7, 95% CI 0.5 to 1.0) and MSVI increased (risk ratio 1.5, 95% CI 1.2 to 0.17).

**Conclusion:**

Significant progress has been made to reduce blindness and increase access to eye health across the Gambia, with further work is needed to decrease the risk of MSVI.

## Introduction

The Gambia is the smallest country on mainland Africa, with an estimated population of 2.3 million (2019).^1^ In 1986, a national survey of vision impairment (VI) provided baseline data for the inception of the National Eye Care Programme, now the National Eye Health Programme (NEHP).^2^ The all-age national prevalence of blindness (visual acuity (VA)<3/60 in the better-seeing eye) was 0.7%; the prevalence of moderate or severe vision impairment (MSVI, VA<6/18 to ≥3/60) was 1.4%.^2^ In 1996, a second national survey evaluated changes in population eye health following 10 years of programme implementation.^3^ Compared with 1986, the national blindness prevalence had fallen to 0.4%, while MSVI prevalence had increased to 1.6%.^3^ The main causes of blindness and MSVI in both surveys were cataract, aphakia, uncorrected refractive error and corneal scarring.^2 3^ However, between the two surveys, the proportion of blindness caused by cataract or trachoma decreased substantially in line with NEHP activities.^2 3^

The NEHP was launched as a three-tier system of primary, secondary and tertiary services.^4 5^ In 1993, a capacity-building programme began training low and mid-grade ophthalmic personnel, with phased implementation from west to east. A comprehensive national Trachoma Control Programme was initiated in 1997, leading to the elimination of trachoma as a public health problem in 2021.^6^

At the community level, primary eye care training is offered to healthcare workers, including community health nurses, village health workers, traditional birth attendants and primary school teachers.^3 7^ Secondary care is delivered through minor and major health facilities, linked by mobile community health nurses. Eight secondary eye units (across all seven health regions) are each led by a senior ophthalmic medical assistant (SOMA), trained in cataract surgery. An optometrist or optometry technician-led vision centre was established in each health region between 2013 and 2019.^8 9^

In 2007, the Sheikh Zayed Regional Eye Care Centre (SZRECC) opened near the capital, expanding ophthalmic training and delivery of ophthalmic services. Subspecialty ophthalmic services comprising paediatrics, glaucoma, medical retina, optometry and orthoptics were introduced through the Gambia–Swansea VISION 2020 Link programme in 2008.^10^ Glaucoma and medical retinal services were further developed in 2017 and are led by a glaucoma specialist. There are presently five full-time ophthalmologists in the Gambia, all stationed at SZRECC.

Changes in population demographics since the last survey include increasing population size, life expectancy and substantial urbanisation.^1 11 12^ Considering these and NEHP service developments, we conducted the third National Eye Health Survey in 2019, to determine the current state of eye health in the Gambia.

## Materials and methods

The purpose of this survey was to determine the prevalence and causes of VI in a nationally representative population-based sample of adults 35 years and older in the Gambia, and to compare this with the situation in 1996.

### Study setting

**Figure 1:**
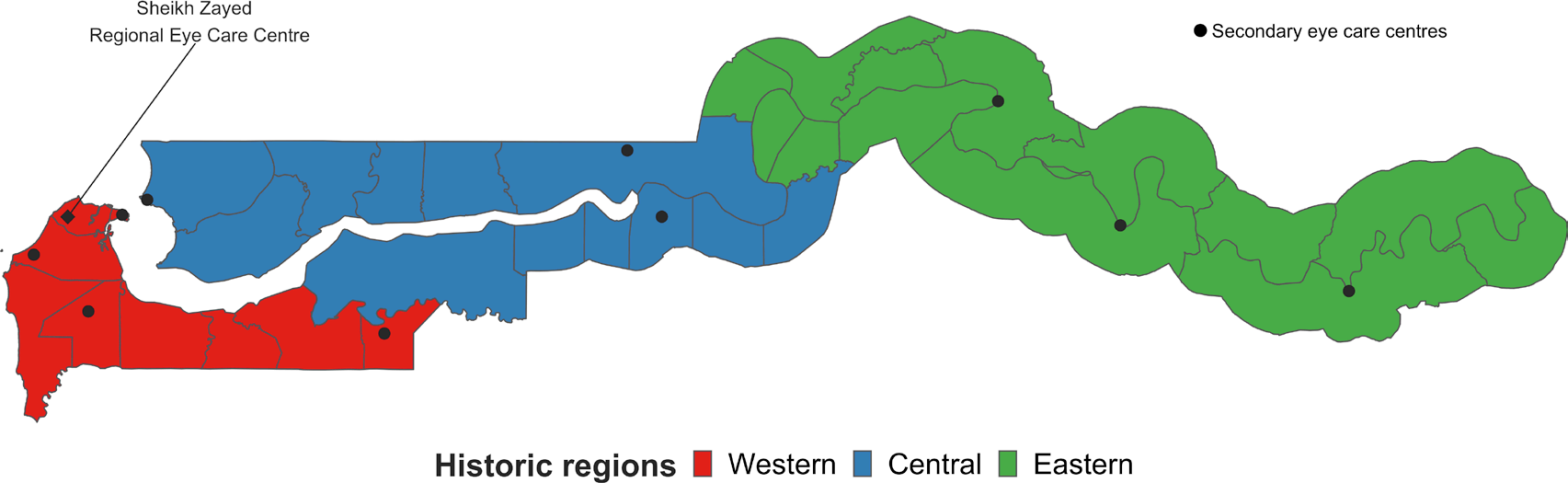
Historic Regions of the Gambia (map generated in the statistical programme R using map data from GADM.org by Ian McCormick)

The Gambia is divided into eight local government areas (LGAs), with 1923 settlements. Since 1996, the administrative boundaries have changed, so to compare findings with previous surveys, we stratified the country into three historic regions: western, central and eastern (figure 1). Western included Brikama, Kanifing and Banjul LGAs. Central included Mansakonko and Kerewan, and eastern included Janjanbureh, Kuntaur and Basse.

### Sampling strategy and sample size

The detailed survey protocol is reported elsewhere.^13^ In brief, we used the most recent available (2013) census data as the sampling frame, and conducted multistage stratified cluster random sampling, to select a nationally representative sample of adults 35 years and older (for comparability with previous surveys). We reviewed eye disease prevalence data from the region and powered the sample size to detect disease prevalence as low as 0.5%.^3 14 15^ We estimated an intraclass correlation coefficient of 0.038, design effect of 2.5, 20% non-response and binomial exact distribution with an estimated margin of error of 0.25% to account for rare conditions (p<0.1) to arrive at an overall sample of 10 800 adults age 35 and older in 360 clusters of 30. We stratified the national sampling frame by urban/rural locality and broad region, selecting 360 clusters without replacement and with probability proportionate to size. Finally, we selected 30 participants per cluster via segmentation (see below).

### Team composition and training

Four teams completed the data collection between February and July 2019. Each included an ophthalmologist, optometrist or optometry technician, SOMA, general nurse, mental health nurse and two enumerators. An audiology nurse joined one of the teams. Teams completed 10 days of training, including an interobserver variability test of VA measurement and pilot testing (detailed elsewhere), before the survey began.^13^

### Data collection procedures

Enumeration Area (EA) maps were used by the enumerators to visit each cluster a day in advance of data collection, to complete a household listing of all eligible residents and identify a central location for the examination. Community members were eligible if they were 35 years or older, had resided in an EA household for at least 6 months of the preceding year, ate shared meals with other household members and did not pay or were not paid by other household members. Following listing, enumerators systematically segmented the list into groups of 30 participants and selected one group at random. Selected participants were given further information about the study and invited to attend the central location the following day.

Data collection procedures not related to this paper are described elsewhere.^13^ The team optometrist or optometry technician measured participants’ VA indoors, without glare affecting either the participant or test chart. Monocular distance VA (uncorrected and wearing available correction) was measured using Peek Acuity.^16^ People with uncorrected (or corrected, if wearing spectacles) VA<6/12 in either eye were retested with pinhole (lorgnette multi 17 occluder) and objective and subjective refraction using a streak retinoscope, trial lens set and wall chart (3 metre Snellen chart, Sussex Vision). We tested monocular best corrected VA with Peek Acuity following refraction. Near vision assessment will be reported separately.

Participants then underwent detailed ocular examination by an ophthalmologist. A desktop-mounted slit-lamp was used to document anterior segment eye disease or trachomatous trichiasis using a standardised eye health survey examination form, comparable with the 1996 data collection.^13^ Unless contraindicated by IOP≥35 mm Hg (measured via iCare ic100 Tonometer) or van Herrick’s grade 2 or 1, both pupils were then dilated using tropicamide 1%, and a slit lamp and a 90 D lens were used to examine the lens and posterior segment.

Images were taken of the anterior (Nikon D5600 Digital camera with macro lens), and posterior segment (disc-centred and macula-centred, using the Remidio Retinal Camera).^17^

### Outcome measures and data analysis

The main outcome measures and definitions are outlined in online supplemental appendix 1 and described in the relevant tables. Details on other study outcome measures, diagnoses and data management, preparation and dissemination are presented in full elsewhere.^13^

10.1136/bjophthalmol-2021-320008.supp1 Supplementary data

Data were analysed using STATA V.16.0. Prevalence estimates with 95% CIs were generated using the ’svyset’ command to account for clustering. The survey oversampled women and older age groups, relative to their proportions in the population in general. Poststratification sample weights were calculated for 5-year age–sex bands, multiplied by cluster selection probabilities to standardise the findings to the 2013 Gambia census. We also generated sampling weights to standardise the findings to the WHO standard population.^13 18 19^

Age-disaggregated estimates are presented above and below 50 years of age, to align with recent global estimates and outputs from the Rapid Assessment of Avoidable Blindness.^20 21^ To estimate the magnitude of VI and blindness, adjusted prevalence estimates were extrapolated from intercensal population projections provided by the Gambia Bureau of Statistics.^22^ To compare outputs from the current survey to previous surveys, crude prevalence estimates for each were first age standardised to the 1986 sample. We then calculated adjusted risk differences and ratios for 1996 and 2019, using the 1986 estimates as denominators. P values for risk ratios (RRs) were generated via χ^2^ tests.

## Results

We examined 9188 participants out of 11 027 eligible, enumerated participants (response rate 83%). Sample characteristics are reported in detail elsewhere.^13^ In brief, the mean age of the examined sample was 49.6 years (SD) 13.4) and 6478 (70.5%) were female. Response rates were lower than expected in several age groups and clusters. However, the sample from the three regions and the representation of the main ethnic groups reflected the 2013 census distributions, and 5039 (54.8%) lived in urban locations.

Table 1 shows crude and census age–sex weighted prevalence estimates for distance VI and blindness in the population 35 years and older across the Gambia. Estimates weighted using the WHO standard population were similar to the census-weighted estimates, and therefore, we only present crude and census-weighted estimates throughout (see online supplemental appendix 2 for WHO weighted estimates by region). The weighted prevalence of blindness was 1.2% (95% CI) 0.9% to 1.4%); moderate or severe vision impairment (MSVI) was 8.9% (95% CI 8.1% to 9.7%); and mild VI was 3.3% (95% CI 3.0% to 3.8%). The total weighted prevalence of any distance VI was 13.4% (95% CI 12.4% to 14.4%). Extrapolating to the 2019 Gambian population, this gives an estimated 5405 people 35+ who are blind and 60 360 with any distance VI.

**Table 1:** Crude and census-weighted prevalence of vision impairment and blindness among the population aged 35 years and older in the Gambia and its historic regions

	National (n=9188)			Western region (n=5625)			Central region (n=1476)			Eastern region (n=2087)		
	Crude		Age and sex standardised (2013 census)	Crude		Age and sex standardised (2013 census	Crude		Age and sex standardised (2013 census)	Crude		Age and sex standardised (2013 census)
	n	% (95% CI)	% (95% CI)	n	% (95% CI)	% (95% CI)	n	% (95% CI)	% (95% CI)	n	% (95% CI)	% (95% CI)
Vision impairment category*												
No vision impairment (≥6/12)	7861	85.6 (84.8 to 86.3)	86.6 (85.6 to 87.6)	4804	85.4 (84.5 to 86.3)	86.1 (84.8 to 97.2)	1210	82.0 (79.9 to 83.9)	83.8 (80.2 to 86.9)	1847	88.5 (87.1 to 89.8)	89.7 (88.0 to 91.2)
Mild (<6/12 and ≥6/18)	326	3.5 (3.2 to 3.9)	3.3 (3.0 to 3.8)	215	3.8 (3.4 to 4.4)	3.7 (3.2 to 4.3)	63	4.3 (3.3 to 5.4)	4.1 (3.0 to 5.6)	48	2.3 (1.7 to 3.1)	2.0 (1.5 to 2.8)
Moderate (<6/18 and ≥6/60)	726	7.9 (7.4 to 8.5)	7.1 (6.4 to 7.9)	463	8.2 (7.5 to 9.0)	7.5 (6.6 to 8.5)	137	9.3 (7.9 to 10.9)	8.3 (6.2 to 11.1)	126	6.0 (5.1 to 7.1)	5.4 (4.3 to 6.9)
Severe (<6/60 and ≥3/60)	171	1.9 (1.6 to 2.2)	1.8 (1.6 to 2.1)	87	1.5 (1.3 to 1.9)	1.6 (1.2 to 2.0)	42	2.8 (2.1 to 3.8)	2.3 (1.6 to 3.3)	42	2.0 (1.5 to 2.7)	1.9 (1.3 to 2.5)
Blind (<3/60)	104	1.1 (0.9 to 1.4)	1.2 (0.9 to 1.4)	56	1.0 (0.8 to 1.3)	1.2 (0.9 to 1.6)	24	1.6 (1.1 to 2.4)	1.4 (0.9 to 2.3)	24	1.1 (0.8 to 1.7)	1.0 (0.6 to 1.5)
<3/60 to ≥1/60	52	0.6 (0.4 to 0.7)	0.6 (0.4 to 0.8)	29	0.5 (0.4 to 0.7)	0.6 (0.4 to 0.9)	14	0.9 (0.6 to 0.2)	0.8 (0.4 to 0.2)	9	0.4 (0.2 to 0.8)	0.4 (0.2 to 0.8)
<1/60 to light perception	36	0.4 (0.3 to 0.5)	0.4 (0.3 to 0.6)	17	0.3 (0.2 to 0.5)	0.3 (0.2 to 0.6)	8	0.5 (0.3 to 1.1)	0.5 (0.2 to 1.1)	11	0.5 (0.3 to 0.9)	0.4 (0.2 to 0.8)
No light perception	16	0.2 (0.1 to 0.3)	0.2 (0.1 to 0.3)	10	0.2 (0.1 to 0.3)	0.2 (0.1 to 0.4)	2	0.1 (0.0 to 0.5)	0.1 (0.0 to 0.3)	4	0.2 (0.1 to 0.5)	0.2 (0.1 to 0.5)
Any VI (<6/12)	1327	14.4 (13.7 to 15.2)	13.4 (12.4 to 14.4)	821	14.6 (13.7 to 15.5)	13.9 (12.8 to 15.2)	266	18.0 (16.1 to 20.1)	16.2 (13.1 to 19.8)	240	11.5 (10.2 to 12.9)	10.3 (8.8 to 12.0)
Moderate-to-severe VI (<6/18 and ≥3/60)	897	9.8 (9.2 to 10.4)	8.9 (8.1 to 9.7)	550	9.8 (9.0 to 10.6)	9.1 (8.1 to 10.1)	179	12.1 (10.6 to 13.9)	10.6 (8.2 to 13.8)	168	8.1 (7.0 to 9.3)	8.9 (8.1 to 9.7)

*WHO ICD-11 categorisation, based on presenting distance visual acuity in better eye.^34^

VI, vision impairment.

The weighted prevalence of any VI was lower in eastern region (10.3%, 95% CI 8.8% to 12.0%) than central (16.2%, 95% CI 13.1% to 19.8%) or western (13.9%, 95% CI 12.8% to 15.2%) regions. There were no differences in the weighted prevalence of MSVI or blindness between the three major regions but mild VI prevalence was lower in eastern region (2.0%, 95% CI 1.5% to 2.8%) than western region (3.7%, 95% CI 3.2% to 4.3%) or central region (4.1%, 95% CI 3.0% to 5.6%).

The weighted prevalence of any VI and MSVI was higher in women than men (women: any VI 14.0% (95% CI 13.2% to 14.9%), MSVI 11.2% (95% CI 10.2% to 12.4%); men: any VI 10.8% (95% CI 9.7% to 12.0%), MSVI 6.5% (95% CI 5.7% to 7.5%). The prevalence of mild VI and blindness were similar by sex. There were no prevalence differences between rural and urban settings for any VI category. The prevalence of all levels of VI was substantially higher in people aged 50 years and older compared with those 35–49 years (table 2).

**Table 2:** Crude and census-adjusted prevalence of vision impairment and blindness by sex, age group and location

	Male (n=2710)			Female (n=6478)			35–49 (n=3498)			50+ (n=5690)			Rural (n=4149)			Urban (n=5039)		
	Crude		Weighted†	Crude		Weighted†	Crude		Weighted†	Crude		Weighted†	Crude		Weighted†	Crude		Weighted†
	n	% (95% CI)	% (95% CI)	n	% (95% CI)	% (95% CI)	n	% (95% CI)	% (95% CI)	n	% (95% CI)	% (95% CI)	n	% (95% CI)	% (95% CI)	n	% (95% CI)	% (95% CI)
Vision impairment category*																		
No vision impairment (≥6/12)	2289	84.5 (83.1 to 85.8)	86.9 (85.5 to 88.2)	5572	86.0 (85.1 to 86.8)	81.3 (79.8 to 82.7)	4990	96.6 (96.0 to 97.0)	96.6 (95.8 to 97.3)	2871	71.4 (70.0 to 72.8)	72.9 (71.0 to 74.6)	3539	85.3 (84.2 to 86.3)	87.1 (85.4 to 88.6)	4322	85.8 (84.8 to 86.7)	86.2 (85.0 to 87.5)
Mild (<6/12 and ≥6/18)	117	4.3 (3.6 to 5.2)	3.7 (3.1 to 4.5)	209	3.2 (2.8 to 3.7)	4.2 (3.6 to 4.8)	54	1.0 (0.8 to 1.4)	1.2 (0.8 to 1.5)	272	6.7 (6.0 to 7.6)	6.4 (5.7 to 7.3)	148	3.6 (3.0 to 4.2)	3.2 (2.7 to 3.9)	178	3.5 (3.1 to 4.1)	3.4 (2.9 to 4.0)
Moderate (<6/18 and ≥6/60)	201	7.4 (6.5 to 8.5)	6.2 (5.3 to 7.2)	525	8.1 (7.5 to 8.8)	11.0 (9.9 to 12.2)	98	1.9 (1.6 to 2.3)	1.7 (1.3 to 2.3)	628	15.6 (14.5 to 16.8)	14.6 (13.2 to 16.1)	319	7.7 (6.9 to 8.5)	6.7 (5.6 to 8.0)	407	8.1 (7.4 to 8.9)	7.5 (6.5 to 8.5)
Severe (<6/60 and ≥3/60)	58	2.1 (1.7 to 2.8)	1.8 (1.4 to 2.4)	113	1.7 (1.5 to 2.1)	2.4 (2.0 to 2.9)	16	0.3 (0.2 to 0.5)	0.4 (0.2 to 0.7)	155	3.9 (3.3 to 4.5)	3.7 (3.1 to 4.3)	91	2.2 (1.8 to 2.7)	1.9 (1.5 to 2.4)	80	1.6 (1.3 to 2.0)	1.6 (1.3 to 2.1)
Blind (<3/60)	45	1.7 (1.2 to 2.2)	1.4 (1.0 to 1.8)	59	0.9 (0.7 to 1.2)	1.2 (0.9 to 1.6)	10	0.2 (0.1 to 0.4)	0.3 (0.1 to 0.5)	94	2.3 (1.9 to 2.9)	2.4 (1.9 to 3.0)	52	1.3 (1.0 to 1.6)	1.1 (0.8 to 1.5)	52	1.0 (0.8 to 1.4)	1.2 (0.9 to 1.7)
Any VI (<6/12)	421	15.5 (14.2 to 17.0)	10.8 (9.7 to 12.0)	906	14.0 (13.2 to 14.9)	15.9 (14.7 to 17.2)	178	3.4 (3.0 to 4.0)	3.4 (2.8 to 4.2)	1149	28.6 (27.2 to 30.0)	27.2 (25.4 to 29.0)	610	14.7 (13.7 to 15.8)	12.9 (11.5 to 14.6)	717	14.2 (13.3 to 15.2)	13.7 (12.5 to 15.0)
Moderate-to-severe VI (<6/18 and ≥3/60)	259	9.6 (8.5 to 10.7)	6.5 (5.7 to 7.5)	638	9.9 (9.2 to 10.6)	11.2 (10.2 to 12.4)	114	2.2 (1.8 to 2.6)	2.1 (1.6 to 2.7)	783	19.5 (18.3 to 20.7)	18.3 (16.8 to 19.9)	410	9.9 (9.0 to 10.8)	8.6 (7.4 to 10.0)	487	9.7 (8.9 to 10.5)	9.1 (8.1 to 10.2)

*WHO ICD-11 categorisation, based on presenting distance visual acuity in better eye.^34^

†Weighted to age–sex distribution of the Gambia 2013 census.

VI, vision impairment.

To report temporal trends, table 3 presents the crude national prevalence of blindness and MSVI (termed ‘low vision’ in earlier surveys) by age group in 1986, 1996 and 2019. To enable comparisons between surveys, we also provide the prevalence estimates for the 1996 and 2019 surveys, standardised to the 1986 age structure. The relative risk of blindness calculated across the adjusted estimates was similar between 1986 and 1996 (risk ratio (RR) 0.8, 95% CI 0.6 to 1.2) but decreased between 1996 and 2019 (RR 0.7, 95% CI 0.5 to 1.0). The relative risk of MSVI increased between 1986 and 1996 (RR 1.3, 95% CI 1.0 to 1.6), and between 1996 and 2019 (RR 1.5, 95% CI 1.2 to 1.7). Compared with 1986, the 2019 relative risk of blindness was 0.5 (95% CI 0.3 to 0.7), and of MSVI was 1.9 (95% CI 1.6 to 2.3).

**Table 3:** Relative risk of blindness and MSVI nationally by age 1986, 1996 and 2019

	Sample examined			Blindness (crude)			MSVI (crude)		
	1986	1996	2019	1986	1996	2019	1986	1996	2019
Age	**n**	**n**	**n**	**%**	**%**	**%**	**%**	**%**	**%**
30–39†	807	1303	2249	0.4	0.4	0.1	0.9	0.4	1.2
40–49	590	842	3096	1.0	0.2	0.3	1.9	2.1	2.7
50–59	407	496	1741	1.5	1.9	0.6	4.2	6.5	8.0
60–69	255	369	1154	3.9	3.5	1.2	8.6	12.1	18.0
70+	226	278	948	9.7	7.5	7.1	19.9	28.1	37.6
Crude prevalence				2.1 (1.5 to 2.6)	1.5 (1.1 to 2.0)	1.2 (0.9 to 1.4)	4.5 (3.6 to 5.3)	5.4 (4.7 to 6.2)	8.9 (8.3 to 9.5)
Age-standardised prevalence‡				–	1.7 (1.2 to 2.1)	1.1 (0.9 to 1.3)	–	6.0 (5.2 to 6.8)	8.3 (7.9 to 8.8)
Risk ratio (compared with 1986)§				–	0.8 (0.6 to 1.2)	0.5** (0.3 to 0.7)	–	1.3* (1.0 to 1.6)	1.9** (1.6 to 2.3)
Risk difference (compared with 1986)¶				–	−0.004 (−0.011 to 0.003)	−0.009 (−0.015 to −0.003)	–	0.135 (0.002 to 0.025)	0.387 (0.029 to 0.048)
Risk ratio (compared with 1996)§					–	0.7* (0.5 to 1.0)		–	1.5 (1.2 to 1.7)**
Risk difference (compared with 1996)¶					–	−0.005 (−0.010 to −0.000)		–	0.252 (0.016 to 0.346)

NB 1996 and 1986 comparison data generated from unpublished survey data provided by H Faal.

*p<0.05 from χ^2^ test.

†35–39 in 2019.

‡Age standardised to 1986 sample.

§Age-standardised prevalence risk ratio.

¶Age-standardised prevalence risk difference.

**p<0.001 from χ^2^ test.

MSVI, moderate or severe vision impairment.

In 2019, cataract was the main cause of blindness (71.0%), severe VI (67.2%), moderate VI (49.9%) and VI overall (44.6%), while refractive error was the main cause of mild VI (83.3%). More than two-thirds (68.8%) of people with any VI had more than one cause contributing to their vision loss (table 4).

**Table 4:** Main causes of blindness and vision impairment (adjusted number of cases* and proportions by level of vision impairment) in the Gambia in 2019

	Vision impairment (VI) category†								Broad VI category			
	Mild (<6/12 and ≥6/18)		Moderate (<6/18 and ≥6/60)		Severe (<6/60 and ≥3/60)		Blind (<3/60)		Any VI (<6/12)		Moderate-to-severe VI (<6/18 and ≥3/60)	
	N	%	N	%	N	%	N	%	N	%	N	%
Refractive error	256	83.4	236	36.1	6	3.9	1	0.8	498	40.6	242	29.7
Cataract	38	12.4	326	49.8	108	67.2	75	71.0	547	44.6	433	53.3
Aphakia	0	0.0	9	1.3	9	5.7	3	2.9	21	1.7	18	2.2
Trachomatous corneal opacity	0	0.0	1	0.2	0	0	1	1.2	2	0.2	1	0.1
Other corneal opacity	2	0.5	9	1.4	5	3.3	9	8.5	25	2.0	14	1.8
Other anterior segment cause	8	2.5	29	4.4	21	13.4	13	13.1	72	5.9	50	6.2
Posterior segment cause	2	0.7	14	2.2	8	5.2	3	2.6	28	2.2	23	2.8
Globe/central nervous system abnormality	0	0.0	0	10	1	0.6	0	0.0	1	0.1	1	0.1
Unknown	1	0.4	31	4.7	1	0.7	0	0.0	33	2.7	33	3.9
		100.0				100.0		100.0		100.0		100.0
Comorbidities‡	253	82.8	430	68.9	103	64.5	70	65.9	855	71.6	532	68.0

*N and % are based on the weighted sample, after correcting the age/sex imbalance in 2019 using the Gambia 2013 census.

†WHO ICD-11 categorisation, based on presenting distance visual acuity in better eye.^34^

‡The presence of at least 1 of 12 main causes in same person.

## Discussion

The census-weighted prevalence of blindness in adults aged 35 years and older in the Gambia in 2019 was 1.2% (0.9–1.4), while the prevalence of MSVI was 8.9% (8.1–9.7). There have been no comprehensive eye health surveys in sub-Saharan Africa in the last decade with which to compare this estimate.^23^ Meta-analyses by the Vision Loss Expert Group (VLEG) in 2020 estimated the all-age prevalence of blindness in western sub-Saharan Africa at 1.1% (1.0–1.3), and the all-age prevalence of MSVI in the region at 4.1% (3.6–4.5).^24^ These estimates are not directly comparable, due to low prevalence in children and younger adults reducing all-age estimates, compared with those in adult subgroups only. VLEG also estimates that the prevalence of blindness in the population 50 years and older in the region is 4.2% (3.5–4.9) and MSVI is 14.4% (12.7–16.3).^20^ In comparison, our study estimated the weighted prevalence of blindness in this age group as 2.4% (1.9–3.0), and MSVI 18.3% (16.8–19.9).

While prevalence of mild VI was higher in eastern region than other regions, we otherwise detected few differences in the prevalence of each level of VI by broad geographic region or rural vs urban location. The absence of subnational discrepancies in prevalence validates the NEHP’s approach to tiered, regional eye health service provision, including stationing mid-level ophthalmic personnel to perform surgeries throughout the country, and ensuring effective referral between levels.

The prevalence of moderate VI in our survey was higher in women than men (11.0% (9.9–12.2) vs 6.2% (5.3–7.2)) but VI prevalence was otherwise similar by sex. This latter finding is encouraging, given commonly documented gender inequity in accessing eye health services globally.^25^ The 1986 Gambian survey reported an all-age, age-standardised 1.6:1 ratio of VI (<6/18) in women versus men (2.5% vs 1.6%), attributed by the authors predominantly to differences in health-seeking behaviour for cataract and trachoma treatment.^2^ Changing patterns of disease (for example declining contribution of trachoma to VI) and increasing service provision may be responsible for reducing some gender inequality, although the higher estimate for moderate VI in women requires further investigation.

The Gambian population has almost tripled between 1986 and 2019^2 18^ and a parallel rise in life expectancy has increased both the absolute number of older people and the older population as a proportion of the all-age population.^26^ Despite this increase in the population with expected eye health service needs, the relative risk of blindness in 2019 is 30% lower than 1996 (RR 0.7, 0.5–1.0) and has halved compared with 1986 (RR 0.5, 0.3–0.7). In 2020 VLEG estimated a 27.3% (26.4–28.0) reduction in blindness in the region between 1990 and 2019.^24^ The prevalence of MSVI in the Gambia (standardised to the 1986 Census population) increased slightly between 1986 and 1996 (4.5%–6.0%, RR 1.3, 1.0–1.7) but remained similar between 1996 and 2019 (RR 1.3, 1.0–1.6). By comparison, VLEG estimated a 3.4% (2.8–4.0) reduction in age-standardised MSVI in the region over this period.^24^ The NEHP has had a positive impact on population eye health in terms of blindness reduction, but in contrast to regional estimates, has not seen a similar decline in MSVI. Maintaining the impressive progress in treating those most severely impaired while managing the effects of population growth and ageing on an increasing magnitude of moderate and severe impairment will be a planning priority over the coming decades.

Comparisons with the 1986 and 1996 surveys also highlight changes in the proportion of blindness by cause over time. In 2019, 71% of blindness was due to cataract, a reversal of the modest decline in the proportion from 55% to 45% between 1986 and 1996 but in line with recent Global Burden of Disease (GBD) estimates that cataract continues to be the leading cause of blindness in the region.^2 3^ High-volume manual small incision cataract surgery has been performed in the Gambia since 2015, but the current surgical threshold is 6/60 in most facilities (6/18 in SZRECC).^27^ The enduring high proportion of VI attributed to cataract warrants revision of these guidelines, and a focus on further expansion of cataract surgical services.^28^

In contrast, and contributing to the increased proportion of blindness caused by cataract, declining proportions of other avoidable causes of blindness highlights strengths of the NEHP. Trachoma was responsible for 17% of blindness in 1986, 5% in 1996 and 1% (1 case) in 2019.^2 3^ NEHP’s commitment to the SAFE strategy^29 30^ has recently seen the Gambia become only the second sub-Saharan African country to eliminate trachoma as a public health problem. Similarly, uncorrected aphakia was the cause of 8% of blindness in 1986 and 13% in 1996.^2 3^ By 2019, only 2 cases were identified as causes of VI, suggesting improved surgical outcomes with increased use of intraocular lenses.^31^

Despite the achievements of the Regional Ophthalmic Training Programme, there is anecdotal evidence of depleted eye health personnel in community and secondary facilities nationally, which undoubtably affects service coverage overall. Motivation and retention of competent and committed personnel is essential to integrate eye care into universal health coverage, support effective eye care delivery, and achieve universal eye health.^32 33^ Ensuring adequate career progression, availability of programme resources and full integration of services into primary and secondary health facilities are all strategies that can support the NEHP to continue tackling avoidable VI.^32^

This large, comprehensive survey provides rich data on population eye health in the Gambia in 2019, and allows comparison of changes in VI and blindness over time. However, under sampling in certain age groups and clusters necessitated the use of sampling weights in reporting estimates.

In conclusion, the 2019 Gambia National Eye Health Survey demonstrated a reduction in the prevalence of blindness compared with 1986 and 1996. There was no significant difference in the prevalence of VI or blindness across the regions or by urban/rural locations, indicating strong national coverage by the NEHP. Huge progress has been made to achieve the elimination of trachoma as a public health problem in 2021. Now, the volume of quality cataract surgery needs to increase to address the leading cause of blindness and MSVI. The infrastructure required to avoid subnational eye health inequality appears to be in place, but a national strategy for early intervention via modern cataract surgical services is necessary and targeted interventions to reduce gender inequality in moderate impairment may be needed.

## Data Availability

Data are available upon reasonable request. Data analyses are ongoing. The anonymised dataset can be made available on reasonable request from the study team.
