# Supplementary material for: Prevalence of blindness and distance vision impairment in the Gambia across three decades of eye health programming
Source: Br J Ophthalmol. 2021 Dec 23;107(6):876–82. doi: 10.1136/bjophthalmol-2021-320008 (PMC10314069; doi:10.1136/bjophthalmol-2021-320008)
Supplement: Supplementary data [file bjophthalmol-2021-320008supp001.pdf]

Appendix 1: Definitions of outcome measures

Measure	Category	Definition
Distance Vision Impairment Category[34]	No Vision Impairment	PVA ≥ 6/12 in the better seeing eye
	Mild Vision Impairment	PVA <6/12 and ≥ 6/18 in the better seeing eye
	Moderate Vision Impairment	PVA <6/18 and ≥6/60 in the better seeing eye
	Severe Vision Impairment	PVA <6/60 and ≥3/60 in the better seeing eye
	Blind	PVA <3/60 in the better seeing eye
Sub-categories of blindness[34]	Not blind	PVA ≥ 3/60in the better seeing eye
	<3/60 – 1/60	PVA <3/60 and ≥ 1/60 in the better seeing eye
	<1/60 – Perception of Light (PL)	PVA ≥ 1/60 and light perception in the better seeing eye
	No Perception of Light (NPL)	No light perception in the better seeing eye
Additional Categories used in text	Any Vision Impairment	Presenting distance visual acuity (PVA, with available correction if worn) <6/12 in the better seeing eye
	Moderate to Severe Vision Impairment (MSVI)	PVA <6/18 and ≥3/60 in the better seeing eye; defined as "Low Vision" in 1996 paper

Anterior Segment Eye Disease	Any Refractive Error	Uncorrected visual acuity (UCVA) <6/12 improving to ≥ 6/12 with available correction, pinhole or refraction
	Vision Impairing Refractive Error	PVA<6/12 improving to ≥ 6/12 with pinhole or refraction
	Cataract[17]	Any grade 1 - 3 of nuclear, cortical or posterior capsular cataract or, if ungradable, any cataract marked mature or hypermature using WHO Cataract Grading Tool
	Cataract Surgical Complications	Aphakia, posterior capsular opacification, aphakic bullous or pseudophakic bullous keratopathy identified on ophthalmic examination
	Trachoma corneal opacity[18,19]	Current trichiasis (defined using WHO 2019 definition), or evidence of prior trichiasis surgery alongside corneal scarring (C2a – C4 only) in the same eye
	Other corneal opacity[19]	Corneal scarring but no prior trichiasis or prior trichiasis surgery in the same eye (C2a – C4 only)
	Other anterior segment eye disease	Presence of at least one of the below pre-coded diseases, identified on slit lamp examination: pterygium (cornea involved), band keratopathy, corneal ulcer, uveitis, or other anterior segment ocular disease *or* other anterior segment disease described in open text
Posterior Segment Eye Disease	Age-related maculopathy and degeneration (ARMD)	Any ARMD including: drusen or hypo/hyper pigmentation without degeneration, dry or geographic, or wet/neovascular or disciform
	Glaucoma[20]	99.5% percentile of cup-disc ratio or asymmetry (Category 2), based on field grading. If optic disc not visible: PVA <3/60 and IOP in the 99.5% percentile
	Any diabetic retinopathy[21]	Any diabetic retinopathy at least R1 or M1 using the Scottish Grading System, based on dilated ocular photograph grading
	Sight-threatening diabetic retinopathy (STDR)[22]	Proliferative Retinopathy (R4) or Referable Maculopathy (M2) using the Scottish Grading System, based on dilated ocular photograph grading
	Optic disc atrophy	Optic disc atrophy marked as present but does not meet glaucoma definition
	Other posterior segment (per eye)	Presence of pseudoexfoliation, identified on slit lamp examination *or* other posterior segment disease described in open text
Main cause of distance vision impairment		In all eyes with PVA<6/12, disease presence as above.<br><br>If more than one of the above definitions are met in one eye using the definitions above, the main cause will be listed as the highest ranking in order of:<br>1. Refractive Error<br>2. Cataract

	3. Aphakia<br>4. Trachomatous corneal opacity<br>5. Other corneal opacity<br>6. Other Anterior Segment<br>7. Posterior segment<br>8. Globe<br>9. Unknown<br><br>If more than one of the above definitions is met in one person, the main cause at the person level will be listed as the highest ranking in this order. Participants with PVA<6/12 with no reported anterior or posterior segment disease as defined above were categorised as unknown.<br><br>A known limitation of this hierarchical approach to determining the “main cause” is that it will lead to underestimation of posterior segment causes. The proportion of people with co-morbidities will be reported, and manuscripts detailing prevalence and associations of specific eye diseases will provide further detailed breakdown on anterior and posterior causes of VI.

Appendix 2: Crude and weighted prevalence of vision impairment and blindness among the population aged 35 years and older in The Gambia and its historic regions – age and sex standardised to WHO Reference Population, and the Gambia 2013 Census

	National				Western Region			Central Region			Eastern Region		
	Crude		Age and sex standardised (WHO)	Age and sex standardised (2013 Census)	Crude		Age and sex standardised (2013 Census)	Crude		Age and sex standardised (2013 Census)	Crude		Age and sex standardised (2013 Census)
	n	% (95% CI)	% (95% CI)	% (95% CI)	n	% (95% CI)	% (95% CI)	n	% (95% CI)	% (95% CI)	n	% (95% CI)	% (95% CI)
**Vision impairment category[1]**													
**No Vision Impairment (≥ 6/12)**	7861	85.6 (84.8-86.3)	84.1 (82.9-85.2)	86.6 (85.6-87.6)	4804	85.4 (84.5-86.3)	86.1 (84.8-97.2)	1210	82.0 (79.9-83.9)	83.8 (80.2-86.9)	1847	88.5 (87.1-89.8)	89.7 (88.0-91.2)
**Mild (<6/12 and ≥ 6/18)**	326	3.5 (3.2-3.9)	3.9 (3.5-4.4)	3.3 (3.0-3.8)	215	3.8 (3.4-4.4)	3.7 (3.2-4.3)	63	4.3 (3.3-5.4)	4.1 (3.0-5.6)	48	2.3 (1.7-3.1)	2.0 (1.5-2.8)
**Moderate (<6/18 and ≥6/60)**	726	7.9 (7.4-8.5)	8.6 (7.8-9.5)	7.1 (6.4-7.9)	463	8.2 (7.5-9.0)	7.5 (6.6-8.5)	137	9.3 (7.9-10.9)	8.3 (6.2-11.1)	126	6.0 (5.1-7.1)	5.4 (4.3-6.9)
**Severe (<6/60 and ≥3/60)**	171	1.9 (1.6-2.2)	2.1 (1.8-2.5)	1.8 (1.6-2.1)	87	1.5 (1.3-1.9)	1.6 (1.2-2.0)	42	2.8 (2.1-3.8)	2.3 (1.6-3.3)	42	2.0 (1.5-2.7)	1.9 (1.3-2.5)
**Blind (<3/60)**	104	1.1 (0.9-1.4)	1.3 (1.1-1.6)	1.2 (0.9-1.4)	56	1.0 (0.8-1.3)	1.2 (0.9-1.6)	24	1.6 (1.1-2.4)	1.4 (0.9-2.3)	24	1.1 (0.8-1.7)	1.0 (0.6-1.5)
**Sub-category of blindness**													
**<3/60 to ≥1/60**	52	0.6 (0.4-0.7)	0.6 (0.5-0.8)	0.6 (0.4-0.8)	29	0.5 (0.4-0.7)	0.6 (0.4-0.9)	14	0.9 (0.6-0.2)	0.8 (0.4-0.2)	9	0.4 (0.2-0.8)	0.4 (0.2-0.8)
**<1/60 to light perception**	36	0.4 (0.3-0.5)	0.5 (0.3-0.6)	0.4 (0.3-0.6)	17	0.3 (0.2-0.5)	0.3 (0.2-0.6)	8	0.5 (0.3-1.1)	0.5 (0.2-1.1)	11	0.5 (0.3-0.9)	0.4 (0.2-0.8)
**No light perception**	16	0.2 (0.1-0.3)	0.2 (0.1-0.4)	0.2 (0.1-0.3)	10	0.2 (0.1-0.3)	0.2 (0.1-0.4)	2	0.1 (0.0-0.5)	0.1 (0.0-0.3)	4	0.2 (0.1-0.5)	0.2 (0.1-0.5)
**Additional categories**													
**Any VI (<6/12)**	1327	14.4 (13.7-15.2)	15.9 (14.8-17.1)	13.4 (12.4-14.4)	821	14.6 (13.7-15.5)	13.9 (12.8-15.2)	266	18.0 (16.1-20.1)	16.2 (13.1-19.8)	240	11.5 (10.2-12.9)	10.3 (8.8-12.0)
**Moderate to severe VI (<6/18 and ≥3/60)**	897	9.8 (9.2-10.4)	10.7 (9.8-11.7)	8.9 (8.1-9.7)	550	9.8 (9.0-10.6)	9.1 (8.1-10.1)	179	12.1 (10.6-13.9)	10.6 (8.2-13.8)	168	8.1 (7.0-9.3)	8.9 (8.1-9.7)

[1]World Health Organisation ICD-11 Categorisation, based on presenting distance visual acuity in better eye
